# Dosimetry model for photobiomodulation based on anthropometric and hemodynamic variables in patients with orofacial pain post-Covid-19: Study protocol for randomized clinical trial

**DOI:** 10.1371/journal.pone.0309073

**Published:** 2024-10-15

**Authors:** Silvana Simões Velloso Schuler, Mayra Costanti Vilela Campos, Ana Julia Lacerda, Adriana Cátia Mazzoni, Tamiris Silva, Francine Cristina da Silva, Manoela Domingues Martins, Kristianne Porta Santos Fernandes, Elsa Susana Fonseca, Raquel Agnelli Mesquita-Ferrari, Anna Carolina Ratto Tempestini Horliana, Sandra Kalil Bussadori, Lara Jansiski Motta

**Affiliations:** 1 Nove de Julho University, UNINOVE, São Paulo, SP, Brazil; 2 Federal University of Bahia, Vitória da Conquinta, BA, Brazil; 3 Federal University of Rio Grande do Sul, Porto Alegre, RS, Brazil; 4 Universidade da Beira Interior, Covilhã, Portugal; Massachusetts General Hospital, UNITED STATES OF AMERICA

## Abstract

**Introduction:**

Orofacial pain and tension headache are symptoms that affect a large portion of the population, compromising productivity, social ability, and functional development. The treatment for reducing painful sensation should be chosen carefully, as pharmacological treatment may bring side effects and overload the organism of patients in pain. Low-level laser therapy has been used with local and systemic [vascular] applications for pain control. However, there is still uncertainty in the literature about the ideal dosimetric parameters for photobiomodulation treatment according to patient characteristics.

**Methods:**

The objective of this project is to validate a dosimetry model based on the relationship between the effects of photobiomodulation with anthropometric and hemodynamic variables, both in local application and systemic application in patients with symptoms of orofacial pain and tension headache. For this purpose, 180 participants with orofacial pain post-covid eligible participants will be randomly assigned to Group 1—Local Photobiomodulation, Group 2—Vascular Photobiomodulation, Group 3—Placebo Local Photobiomodulation, or Group 4—Placebo Vascular Photobiomodulation [Therapy EC–DMC device, São Carlos, Brazil,– 660 nm, 100mW] using stratified block randomization. Before the application, sociodemographic information such as age, skin phototype [classified by the Fitzpatrick scale], weight, height, body mass index [BMI], oxygen saturation [SaO2], blood pressure [BP], heart rate [HR], and thickness of skin, fat, and facial muscles will be collected. During the application, we will collect local temperature, SaO2, BP, and HR. Before and after laser application, blood levels of lactate and hemoglobin, BP, and HR will be measured in the first and last session. In addition to demographic, anthropometric, and hemodynamic variables, the penetrated energy will be quantified using a power meter, and information from orofacial pain and headache symptom questionnaires will be analyzed. The Monte Carlo simulation technique will be used to systematically study the relationship between the light penetration profile into the target tissues and the most relevant variables, namely BMI, tissue layer thicknesses, and skin phototype. Light transmittance, measured in vivo and simulated, will be compared to validate a personalized dosimetry model.

**Discussion:**

The results of this study contribute to validating a Monte Carlo Simulation model to calculate the appropriate dosimetry for photobiomodulation therapies in the control of patients with Post-Covid-19 orofacial pain.

**Trial registration:**

**Trial registration number**: NCT06065969.

## Introduction

Orofacial pain has been classified according to its location as dental, periodontal, osseous, muscular, neural, in oral mucosa, in salivary glands, and in the temporomandibular joint [1, 2]. It may also present similar manifestations to primary headaches, be of idiopathic origin, or be associated with psychological [anxiety, catastrophizing, and depression] and social factors [access to medical care, stigmas, and support from family and friends] [3, 4]. Pain of dentoalveolar origin and associated structures is the most common complaint in the orofacial region [5, 6] with non-odontogenic pain originating from temporomandibular disorder [TMD] being the most diagnosed in this region [3, 7].

The treatment plan for orofacial pains should be tailored to the individual’s needs. Different therapies can assist in reducing the painful sensation in this context. Caution is advised in the choice of invasive therapies and irreversible treatments as first-line therapies [8, 9].

Occlusal splints, ultrasound, manual physiotherapy, drug therapy, transcutaneous electrical oral stimulation, and photobiomodulation [PBM], they are among the non-invasive treatments that have been used to alleviate orofacial painful conditions [10]. Low-level laser therapy, as a form of photobiomodulation, has been used as an alternative treatment due to its non-invasive and safe nature, related to its characteristics of low-intensity energy and wavelength, and its presentation of anti-inflammatory, analgesic, and other therapeutic biological responses [11]. The mechanism of PBM may be associated with its influence on the synthesis, release, and metabolism of various substances related to pain and analgesia [9].

In the systematic review and meta-analysis [11] out of 85 articles published between 2009 and 2020, 8 randomized clinical trials were evaluated, comparing photobiomodulation treatment with the placebo group in 181 participants with myofascial orofacial pain. The results were satisfactory for controlling painful sensations; however, the authors highlighted the significant variability in equipment and dosimetric parameters. The difference in the choice of energy, power, and wavelength among the included studies does not allow for the development of an ideal dosimetric protocol for treatment.

In another systematic review published in 2022 [10], the objective was to determine which dosimetric parameters of photobiomodulation provide better effects in reducing pain in patients with orofacial pain. The authors observed that the parameters of wavelength, energy, time, and irradiance were quite different among the included clinical trials. The use of diode or gallium-aluminum-arsenide [GaAlAs] lasers, with wavelengths ranging from 400–800 nm or 800–1500 nm, and energy densities below 25 J/cm^2^ has been documented [10]. For patients with joint pain, diode laser and wavelength between 400 and 800 nm were used. For patients with muscular pain, the following lasers and parameters were used: diode laser, wavelength between 800 and 1500 nm, and 25 J/cm2. For patients with joint and muscular pain, infrared laser, wavelength of 800–1500 nm, 100 J/cm2, and an application time between 15 and 30 s or >60 seconds.

Following this line of research, a clinical trial was conducted to assess the cost-effectiveness of laser therapy in managing facial pain. The research demonstrated that photobiomodulation presented analgesic effects and proved to be more cost-effective compared to placebo and occlusal splint [12].

Clinical trials assessing the effectiveness of photobiomodulation have yielded different conclusions regarding dosimetry, and there remains an uncertainty regarding the development of protocols, considering dosimetric parameters and the justification for choosing such parameters in published works [9].

The delivery of energy during photobiomodulation therapy requires the transmission of photons through outer tissue layers, such as skin and fat, before reaching the desired target. We believe that the thickness of these layers in patients with different body compositions may interfere with the expected results of therapy. However, these data have not yet been explored in clinical trials on this topic [10].

Based on the need for greater dosimetric precision according to individual characteristics, we hypothesize that anthropometric and hemodynamic variables can be used to determine the appropriate parameters for delivering energy as close to the ideal as possible in controlling orofacial pain [12].

Therefore, the aim of this project is to evaluate a Monte Carlo simulation model to predict the distribution of energy and its effects related to anthropometric and hemodynamic variables in photobiomodulation treatment with both local and systemic applications across different age groups [13].

With the development of this study, we expect to create and validate a Monte Carlo Simulation model to calculate the appropriate dosimetry for photobiomodulation therapies in orofacial pain control, according to the physical and physiopathological characteristics of each person. In this way, healthcare professionals can deliver energy closest to the ideal in treatment, achieving desired results, reducing unnecessary exposure time, and avoiding under or over-treatment.

## Materials and methods

### Study location, sample selection, and ethical considerations

This is a clinical study for the validation of a dosimetric model. It received approval from the Research Ethics Committee of Nove de Julho University with protocol number 6.080.655 S2 File. For participants under the age of majority, an assent form will be prepared, along with a consent form for their respective guardians (S1 File). The clinical procedures for recruitment, eligibility assessment, and methodological processes will be conducted at the Integrated Health Outpatient Clinics of UNINOVE–Vergueiro Unit São Paulo, SP, Brazil. Monte Carlo Simulation modeling techniques and statistical analyses will be performed in collaboration with the Department of Physics at the University of Beira Interior Covilhã, Portugal. This study was drafted and planned in accordance with the recommendations outlined in the SPIRIT Standard Protocol Items for Randomized Trials–S3 File statement. The participants will sign a statement of informed consent agreeing to participate in the study. Clinical trial registry: ClinicalTrials.gov Identifier: NCT06065969.

### Sample

Individuals between 7 and 19 years of age with orofacial pain post-covid-19 will be invited to participate in the study. A convenience sample was chosen, totaling 180. Given the lack of definitive studies for sample size calculation regarding persistent COVID-19 symptoms, a convenience sample was used. Participants were chosen based on reported symptoms and inclusion criteria at the university outpatient clinic. This approach was necessary due to the evolving nature of post-COVID-19 symptomatology, which currently prevents standardized sample size determination.

### Inclusion criteria

Individuals at least 1 symptom of pain and/or headache, according to the American Academy of Orofacial Pain (AAOP)questionnaire and the headache questionnaire based on the International Classification of Headache Disorders, 3^rd^ edition (ICHD-3) [14].

### Exclusion criteria

Participants with the following characteristics will be excluded: [14]

Pregnant individuals

Individuals with arrhythmia

Individuals with Thrombocytopenia

Individuals with Sickle Cell Anemia

Individuals with a pacemaker

Individuals with alterations in coagulation factors

History of cancer

History of hyperthyroid

### Discontinuation or interruption criteria

Participants reporting any discomfort during the procedures or showing sensitivity to laser application will be excluded from the analysis, and the procedures will be immediately discontinued upon reporting.

### Study variables

Sociodemographic information such as age, skin phototype [classified by the Fitzpatrick scale], weight and height. The primary outcome is orofacial pain, and the secondary outcomes are oxygen saturation, blood pressure, heart rate, local temperature, blood lactate levels, and hemoglobin levels. In addition to demographic, anthropometric, and hemodynamic variables, the penetrated energy will be quantified using a power meter, and information from orofacial pain and headache symptom questionnaires will be analyzed.

### Randomization

The participants will be informed that they will be randomly assigned to one of the four study groups that will be compared. To allocate the participants to the groups, a stratification process will be carried out based on the answers to the questionnaires and the presence of orofacial pain symptoms and the presence of headache symptoms will be considered strata. Stratification will be carried out to balance the allocation of participants in each group according to stratum.

After stratification, participants will be randomized in a 1:1:1:1 ratio into the 4 groups. The randomization list will be generated by an independent researcher in random blocks of four, electronically via the ramdomizer.org website and kept in opaque envelopes. The allocation will only be revealed to the operating researcher at the time of the intervention. A study team member, separate from the researcher who will administer the intervention, will follow the random allocation sequence to assign participants to a study group and prepare study equipment and materials. Eligible participants will be randomly assigned to Group 1—Local Photobiomodulation, Group 2—Vascular Photobiomodulation, Group 3—Placebo Local Photobiomodulation, or Group 4—Placebo Vascular Photobiomodulation using stratified block randomization generated by the study statistician.

### Data collection

#### Blood lactate

Blood lactate will be collected before treatment, immediately after the first session, and after the last session. The collection will be done at the distal phalanx of the middle finger [one drop of blood], after local hygiene with 70% alcohol. For puncture, the researcher will use surgical gloves and disposable lancets. The blood sample will be analyzed by LACTATE DETECT TD-4261 [Eco Diagnóstica, Nova Lima, MG, Brazil] [10].

#### Hemoglobin level

The hemoglobin level will be analyzed before treatment, immediately after the first session, and after the last session. The same puncture performed for lactate collection will be used for hemoglobin analysis. The blood drop will be analyzed by the Hb Analyzer ECO CARE [Eco Diagnóstica, Nova Lima, MG, Brazil].

The variables of heart rate, blood pressure, oxygen saturation, light transmission, and local temperature will be collected during the application of photobiomodulation, which will take place in a private setting in the dental office of the Health Sector Nucleus of the Teaching Center. The Handheld–Vital Sign Monitor will be used for collecting these variables.

#### Light transmission

The SPER Scientific Pocket Laser Power Meter device [Scottsdale, AZ, USA] will be used. Light transmission will be assessed through the index finger and cheek [masseter] of each participant. The sensor of the equipment will be positioned on the inner region of the cheek at the same time the researcher is applying the laser on the masseter region [2 points].

#### Thickness of the fat and muscle layer of the face

The BodyMetrix 2000 device, a linear ultrasound with a depth of 60mm, will be used to assess the thickness of the facial muscles. The method is safe, emits no radiation, and is non-invasive. During the assessment, the participant will remain in a comfortable position, and a conducting gel will be applied to the skin over the masseter. The device will be slid from the origin to the insertion of the muscle, aiming to obtain precise information. The ultrasonographic images generated will be subsequently analyzed using the BodyView software, for thickness of tissue layers in the facial region.

### Interventions

#### Group 1 –Local photobiomodulation

The treatment will be administered over 2 weeks, totaling 4 sessions [2 sessions per week] with an average duration of 6 minutes per session.

Participants will be positioned in a clinical chair for the application of photobiomodulation. The interventions will be conducted by a trained professional. Group 1 will receive photobiomodulation using the Therapy EC–DMC device, with a red wavelength of 660 nm and power of 100 mW, properly calibrated, delivering 6J per point [60 seconds] at 2 points in the masseter muscle region, 1 point in the left temporal muscle,1 point in the right temporal muscle, 1 point in the left trapezius muscle in the cervical region, 1 point in the right trapezius muscle. The total application time will be 4 minutes per session.

At the time of application, only the participant receiving the treatment, the researcher responsible for the treatment, and the guardian will be present. Everyone will wear specific goggles for eye protection. The equipment’s tip will be disinfected with 70% alcohol and covered with disposable transparent plastic [PVC] to prevent cross-contamination, while the facial area to be irradiated will be cleaned with a 0.2% Chlorhexidine solution. During the applications, the participant will remain seated, with the Frankfurt plane parallel to the ground. Energy penetration will be analyzed at one of the points on the masseter muscle.

#### Group 2 –Vascular photobiomodulation

Vascular Photobiomodulation [VBMP] will be applied using the same device, with an infra-red wavelength of 808 nm and 100 mW, directing the light beam to the radial artery region for 10 minutes per session.

The participant will be seated in a comfortable chair with lateral support for resting their arms during the application. At the time of application, only the participant receiving the treatment, the researcher responsible for the treatment, and the guardian will be present. Everyone will wear specific goggles for eye protection. The active part of the bracelet will be covered with disposable transparent plastic [PVC] to prevent cross-contamination, and for hygiene reasons, the area to be irradiated will be pre-cleaned with a 0.2% Chlorhexidine solution. During the applications, the participant will remain seated, with the Frankfurt plane parallel to the ground. A new treatment method will be administered for 2 weeks, totaling 4 sessions [2 sessions per week] with an average duration of 10 minutes per session.

#### Group 3 –Placebo local photobiomodulation

Group 3 will undergo the same procedures described for Group 1, but the equipment will emit only the sound signal, exactly like the equipment used in Group 1, without the emission of laser light.

#### Group 4 –Placebo vascular photobiomodulation

Group 4 will undergo the same procedures described for Group 2, but the equipment will emit only the sound signal, exactly like the equipment used in Group 1, without the emission of laser light.

**Fig 1:**
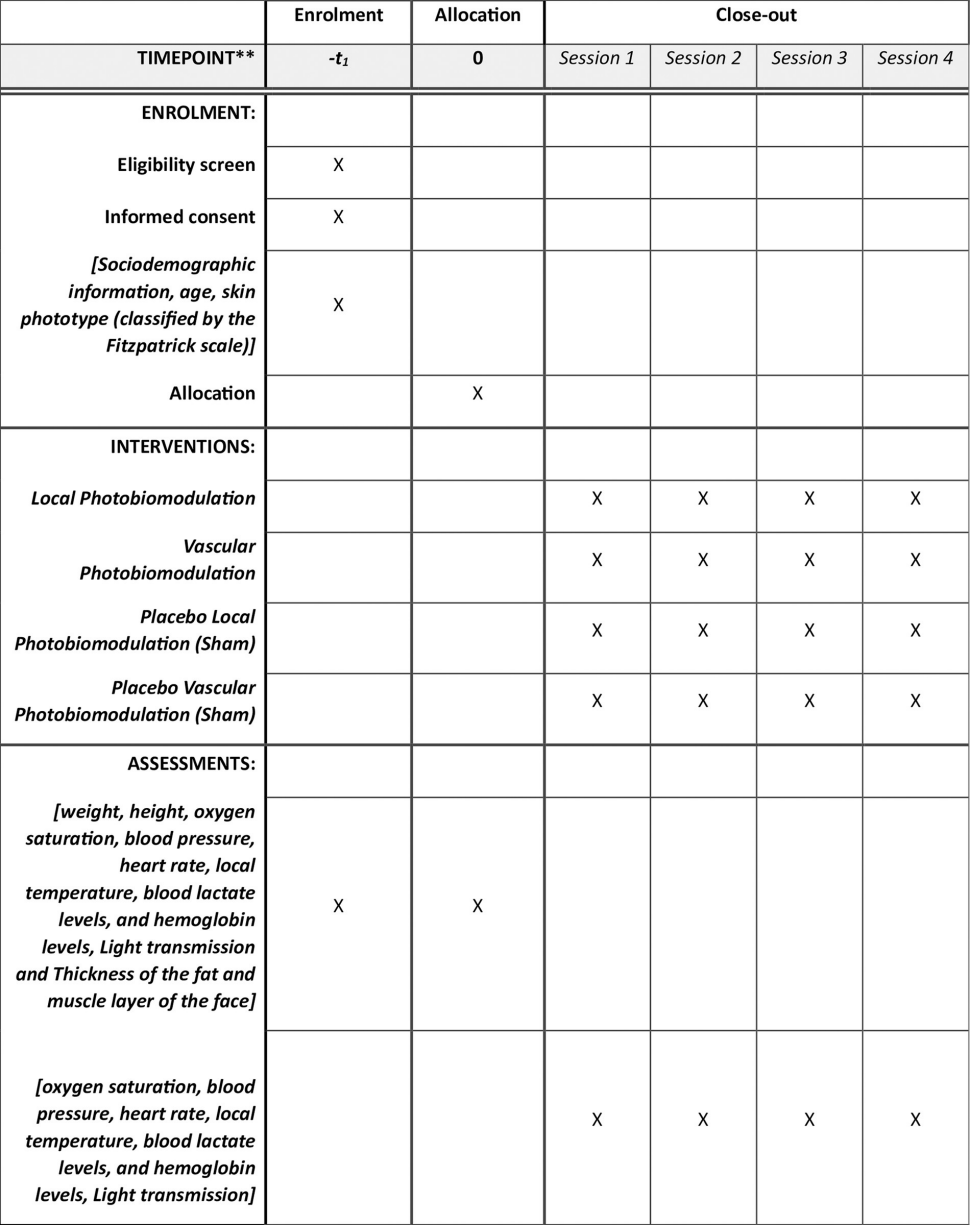
The SPIRIT schedule of enrolment, interventions, and assessments.

The dosimetric parameters for the treatments in Groups 1 and 2 are presented in Table 1. The SPIRIT schedule of enrollment, interventions, and assessments are presented in Fig 1.

**Table 1:** Dosimetric parameters for photobiomodulation application.

PARAMETERS	RED LASER [Local]	INFRARED LASER [Transcutaneous Systemic]
Wavelength [nm]	660	808
Mode	Contínuo	Contínuo
Power [mW]	100	100
Aperture Diameter [cm]	0,354 [beam diameter with spacer]	0,354 cm
Beam Area [cm^2^]	0,0984 [with spacer]	0,0984 cm^2^
Exposure Time [s]	60 per point	600 s
Fluence [J/cm^2^]	61	-
Energy [J]	6 per point	60 J
Number of irradiated points	6	Systemic
Application Technique	Contact	Contact
Number of sessions	4	4
Treatment Frequency	2 times per week	2 times per week
Total Irradiated Energy [J]	144 J	240 J

#### Laser safety measures

The appointments will take place in a dental office, which will have its door closed, preventing people from entering during the use of the laser. Everyone will wear specific glasses for eye protection.

#### Monte Carlo simulation

The Monte Carlo technique will be employed to simulate the fluence profile, energy deposited in different tissue layers of the cheek and index finger, and transmittance, using light of wavelengths 660 nm and 808 nm. In an initial phase, a Monte Carlo model will be developed, considering the morphological structure of the tissues to be treated, approximating their structure to a planar and laminar model consisting of four layers: epidermis, dermis, subcutaneous fat, and muscle [15].

The thicknesses of each layer will be obtained from the literature, and their values will be varied in predefined intervals consistent with literature findings for different age groups and BMI values. The optical properties of each layer, including absorption and scattering coefficients, anisotropy coefficient, and refractive index, will be gathered from bibliographic research, considering the expected variability for the sample under study.

A model for the absorption coefficient will be used, accounting for blood content in each layer, blood oxygen saturation level, as well as fractions of fat, melanin, and water in each tissue layer. Based on the preliminary study, a database will be established aiming to establish a relationship between energy deposited in tissues and different anthropometric characteristics [13, 16].

Special attention will be given to the relationship between energy deposited in the epidermis and muscle, to establish a compromise between safety limits and treatment effectiveness. Simulated transmittance values will be subsequently compared with values measured in volunteers, considering measurements of tissue thickness by ultrasound, skin type, BMI, and blood saturation levels. A computational implementation of the tissue optical model and the Monte Carlo simulation model will be developed based on Marti et al.’s MCMatlab code, available online [https://github.com/ankrh/MCmatlab], using Matlab® software, version 2022.

### Ethical considerations and dissemination

This study will strictly adhere to the ethical principles outlined in the Declaration of Helsinki [World Medical Association Declaration of Helsinki, 2008]. The research protocol has been granted approval by the Ethics Committee Nove de Julho University with protocol number 6.080. 655.Any modifications to the protocol will be promptly communicated to this same committee and duly updated on ClinicalTrials.gov.

Participants will be comprehensively briefed on all potential risks associated with their involvement in the study, as well as the stringent confidentiality measures in place for their data.

The data collected during the research will be stored and organized in the Harvard Dataverse repository [https://dataverse.harvard.edu]. Metadata will be published on the repository’s website through the provided platform link [DOI]. Participant-related data and research outcomes will remain confidential. Only the researchers will have access to this information. After the research is concluded, the data will be published and disseminated in national and international scientific events and journals. Raw data will remain on the platform and, after publication, may be made available to other researchers upon contact with the responsible researcher and under a confidentiality and intellectual property agreement.

### Patient and public involvement

Eligible individuals will receive comprehensive explanations about the study’s aims and procedures. They will provide consent, which will be both written and verbally confirmed. Patients will not participate in recruitment or trial implementation. After data analysis, volunteers may attend a meeting to receive the results. Any potential difficulties arising from the interventions will be evaluated by the patients. The confidentiality of all individuals will be safeguarded throughout the research process. Participants will be allowed to withdraw from the study at any time.

### Expected results

It is expected that we will ascertain whether there is a difference between the effects of vascular and local photobiomodulation on orofacial pain, with the study’s aim to develop and validate a dosimetric model for individualized protocols based on the unique characteristics of each patient.

## Discussion

The present study addresses a significant gap in the understanding of optimal dosimetry for photobiomodulation therapy in patients experiencing orofacial pain and tension headache post-Covid-19. The introduction of low-level laser therapy [LLLT] as a potential alternative for pain management underscores the importance of determining precise dosimetric parameters tailored to individual patient characteristics. This study’s objective to validate a dosimetry model based on anthropometric and hemodynamic variables is crucial for optimizing treatment efficacy and minimizing potential adverse effects associated with pharmacological interventions [9, 10].

An essential aspect of this research lies in its methodological approach, which involves a randomized assignment of participants to different treatment groups, including placebo controls. Such a design ensures rigorous evaluation of the effects of both local and systemic photobiomodulation therapies, thereby enhancing the reliability and validity of the findings. Furthermore, the comprehensive data collection process, encompassing demographic, anthropometric, and hemodynamic variables, as well as measurements of skin and tissue characteristics, enables a thorough investigation of the factors influencing treatment outcomes.

The study’s incorporation of Monte Carlo simulation represents a novel and sophisticated analytical technique for elucidating the relationship between light penetration profiles and key variables, such as BMI and tissue layer thicknesses. By systematically exploring these associations, the research aims to develop a personalized dosimetry model, thereby advancing the precision and effectiveness of photobiomodulation therapy.

The discussion also highlights the potential implications of the study findings for clinical practice, particularly in the context of managing orofacial pain in post-Covid-19 patients. Given the growing recognition of the long-term effects of Covid-19 on various physiological systems, including pain perception and modulation, the validation of a dosimetry model tailored to this population is of paramount importance.

Moreover, the collaborative nature of the study, involving multiple centers across different regions, reflects a concerted effort to address a pressing clinical need in a global context. The prompt ethical approval and enthusiastic participation from the vascular community underscore the study’s relevance and potential impact on improving patient care.

In conclusion, the study’s methodological rigor, innovative approach, and clinical relevance position it as a significant contribution to the field of photobiomodulation therapy. By elucidating the optimal dosimetry parameters for pain management in post-Covid-19 patients, the research has the potential to enhance treatment outcomes and alleviate the burden of orofacial pain and tension headache in this population.

## Supporting information

S1 File Declaration of consent for participation in clinical research original. (PDF)

S2 File Project submitted to the ethics committee [Original Portuguese]. (PDF)

S3 File SPIRIT. This is the S4 File. (PDF)

S4 File Protocol [English]. (PDF)
