# Supplementary material for: Dosimetry model for photobiomodulation based on anthropometric and hemodynamic variables in patients with orofacial pain post-Covid-19: Study protocol for randomized clinical trial
Source: PLoS One. 2024 Oct 15;19(10):e0309073. doi: 10.1371/journal.pone.0309073 (PMC11478869; doi:10.1371/journal.pone.0309073)
Supplement: S1 File — (PDF) [file pone.0309073.s001.pdf]

INFORMED CONSENT FORM
Participant's Name:________________________
Address____________________________Contact Phone:________
City:________ State: __________ Code: __________
E-mail:____________________________

The participant, under their responsibility, is being invited by me, Silvana Simões Velloso Schuler, to participate in a study entitled: **Dosimetry Model For Photobiomodulation Based On Anthropometric And Hemodynamic Variables In Patients With Orofacial Pain Post-Covid-19: Randomized Clinical Trial** The information about the research is as follows:

Experimental Work Title: DOSIMETRIC MODELING FOR PHOTOBIMODULATION BASED ON ANTHROPOMETRIC AND HEMODYNAMIC VARIABLES IN PATIENTS WITH OROFACIAL PAIN POST-COVID-19: STUDY PROTOCOL FOR A RANDOMIZED CLINICAL TRIAL.

Objective: To analyze the relationship between the effects of low-level laser therapy (photobiomodulation) for facial and headache pain and measures such as weight, height, oxygen saturation (SaO2), blood pressure (BP), heart rate (HR), body temperature, and skin phototype.

Rationale: Low-level laser therapy, or photobiomodulation, has been widely used for facial and headache pain in adults, children, and adolescents. Although the treatment is known to reduce pain, it remains unclear whether the light dosage should be proportional to each adolescent's characteristics such as weight, height, blood pressure, heart rate, temperature, skin pigmentation, lactate, and hemoglobin levels.

Experimental Phase Procedures:
The research will be conducted at the integrated health outpatient clinic of the Vergueiro unit of Nove de Julho University, in the city of São Paulo - SP, Brazil.
The participant will be evaluated by the researcher, Pediatric Dentist Silvana Simões, and an assistant from the sector in a private environment, that is, one participant at a time, accompanied by a guardian if desired. We will ask the participant about their health and personal data. Initially, the participant will answer two questionnaires in a private room with only their presence and that of the researcher. One questionnaire has 10 questions, and the other has 13 questions, taking about 10 minutes to answer. There are no right or wrong answers. The participant can choose not to answer any question if they do not want to or do not feel comfortable.
Afterwards, in the participant's presence, we will collect some measurements in a separate room, including weight, height, blood pressure, lactate, and hemoglobin levels.
After that, we will apply the laser light. We will randomly select the participant to receive the light in the painful area of the face/neck or through a bracelet. The participant may be assigned to one of 4 groups: (1) light application on the face or neck; (2) light application through a bracelet, (3) in this group, we will only simulate the light application on the face and neck with the device turned off, and (4) in this group, we will apply the light turned off on the bracelet.

The participant will wear protective glasses, similar to sunglasses, to protect their eyes from the light. This light does not cause any discomfort. It may warm up a little, but it is rare, and if it happens, the participant just needs to let us know, and we will turn off the light immediately.
During the laser light application, we will measure oxygen saturation with an oximeter and the temperature at the application site with a digital thermometer.

In our research, we will meet 4 times (twice a week for two weeks) for laser light application. However, weight and height will only be measured the first time. Blood will be collected only twice, in the first and last sessions. The questionnaires will be answered again in the last session.
If the participant is assigned to the face and neck laser group, the light application will take 18 minutes. If assigned to the bracelet group, they will use the bracelet for 10 minutes. The groups with the devices turned off will take the same respective time.

At the end of the research, if the participant wishes, they can receive the treatment from the group if we discover together that it was better for them.

Expected Discomfort or Risks: The participant may feel embarrassed to answer some questionnaire questions or about their weight and height. The laser poses a risk to the eyes if applied directly to them. The light may cause slight heating at the application site, and blood collection from the finger may cause slight discomfort during collection.
Protective Measures against Risks: We will use specific protective glasses to prevent any damage to the eyes. The laser will be applied by a qualified researcher to ensure safe application. If there is a sensation of heating, as reported by the participant, the equipment will be immediately turned off, and the treatment suspended. To reduce the possibility of embarrassment in answering the questionnaires or in weight and height measurements, these procedures will be carried out in a private room. As for possible discomfort during blood collection, vibration will be used at the site to minimize the discomfort of the needle puncture.
Research Benefits: Laser treatment may alleviate facial and headache pain, as demonstrated in previous research. (Resolution CNS No. 466/12 - Item V and Resolution CNS No. 510/16 - Chapter IV)
Existing Alternative Methods: The existing alternative treatments are pharmacological treatments.
Withdrawal of Consent: The guardian can withdraw their consent at any time without any problem for you or for the adolescent for whom you are responsible. You are free to decide whether you want the adolescent to continue, if you don't like it, don't feel well, and/or it's not what you imagined.
Confidentiality Guarantee: You can rest assured that the personal data we collect from the adolescent will never be disclosed. Information related to this research will only be accessible to authorized individuals such as the researcher and their assistant. However, if any information is disclosed in a report or publication, it will be done in code form to preserve the adolescent's identity and maintain confidentiality. The material will be the responsibility of the principal investigator for a period of 5 years, and after that time, it will be destroyed. Regardless of participation in this research, you may choose to allow the use of the adolescent's image. If you agree, you will have to sign the Image Authorization Form. Otherwise, the photos cannot be used.
"
Compensation for Expenses Resulting from Participation in the Research: You and the adolescent will not receive any monetary compensation for participating in this research, and you will not incur any expenses for treatment. If there is any damage resulting from the research, you have the right to request compensation through legal channels (Resolution CNS No. 510/16, Article 17, Item IX).
Research Location:
Participants will be seen at the integrated health outpatient clinic of the Vergueiro unit of Nove de Julho University, at Rua Vergueiro, 235/249, Liberdade, São Paulo - SP, 01525- 000, Brazil. Email: odontopediatrasilvanasimoes@gmail.com. Contacts: (83)32138714 or (83) 999826708 (direct contact with the researcher).
If you have any questions about the research participant's rights, you can also contact the Research Ethics Committee on Human Beings (CEP/UNINOVE) of Nove de Julho University, by phone (11) 3385-9010. The Research Ethics Committee (CEP) is an interdisciplinary and independent collegiate body that must exist in institutions conducting research involving human

subjects in Brazil, created to defend the interests of research participants in terms of their integrity and dignity, as well as to contribute to the development of research within ethical standards (Regulatory Standards for Research Involving Human Subjects - Res. CNS No. 466/12 and Res. CNS 510/2016). The Ethics Committee is responsible for the evaluation and monitoring of research protocols regarding ethical aspects. Address of Uninove's Ethics Committee: Rua. Vergueiro nº 235/249 - 12th floor - Liberdade - São Paulo - SP CEP. 01504-001. Phone: (11) 3385-9010. Email: comitedeetica@uninove.br. Ethics Committee service hours: Monday to Friday - From 11:30 am to 1:00 pm and from 3:30 pm to 7:00 pm.
Full name and contact phones of the researchers for contact:
Prof. Dr. Lara Jansiski Motta - (011) 998829511 (advisor)
Silvana Simões Velloso Schuler - Contacts: (83) 32138714 or (83) 999826708 (master's student)
Rua Vergueiro, 235/249 - 3rd basement - Liberdade - São Paulo - SP CEP. 01504-001. Email: larajmotta@uni9.pro.br. Email: odontopediatrasilvanasimoes@gmail.com.
Any incidents that may arise during the research can be discussed through the appropriate channels.
São Paulo, on the day of of .

Post-Information Consent:
I,____ , after reading and understanding this information and consent form, understand that my participation is voluntary and that I can withdraw from the study at any time without any harm. I confirm that I have received a copy of this consent form and authorize the conduct of the research work and the disclosure of the data obtained only in this study and in the scientific community.

____
Participant's Signature
(All pages must be initialed by the research participant)

I, (researcher responsible for this study), certify that:
a) This research will only begin after the approval of the Ethics Committee(s) to which the project was submitted.
b) Ethics in research implies respect for human dignity and due protection for the participants of scientific research involving human subjects;
c) This study has scientific merit, and the team of professionals duly mentioned in this form is trained, skilled, and competent to perform the procedures described in this form.

____
Silvana Simões Velloso Schuler
Signature of the responsible researcher

____
Lara Jansiski Motta
Advisor's Signature
