# Supplementary material for: Dosimetry model for photobiomodulation based on anthropometric and hemodynamic variables in patients with orofacial pain post-Covid-19: Study protocol for randomized clinical trial
Source: PLoS One. 2024 Oct 15;19(10):e0309073. doi: 10.1371/journal.pone.0309073 (PMC11478869; doi:10.1371/journal.pone.0309073)
Supplement: S2 File — (PDF) [file pone.0309073.s002.pdf]

VALIDAÇÃO DE MODELO PARA DOSIMETRIA DE FOTOBIOMODULAÇÃO BASEADO EM VARIÁVEIS ANTROPOMÉTRICAS E HEMODINÂMICAS EM PACIENTES COM DOR OROFACIAL: study protocol

Resumo

A dor orofacial e a cefaleia tensional são sintomas que acometem grande parte da população comprometem a produtividade, habilidade social e desenvolvimento funcional. O tratamento para redução da sensação dolorosa deve ser escolhido com cautela, pois o tratamento medicamentoso pode trazer efeitos colaterais e sobrecarregar o organismo dos pacientes com dor. O laser de baixa intensidade tem sido utilizado com aplicações locais e sistêmica (vascular) para o controle da dor. No entanto, há ainda uma dúvida na literatura sobre os parâmetros dosimétricos ideais para o tratamento de fotobiomodulação de acordo com as características do paciente. O objetivo deste projeto é validar um modelo para dosimetria a partir da relação entre os efeitos da fotobiomodulação com as variáveis antropométricas e hemodinâmicas tanto no uso com a aplicação local quanto com a aplicação sistêmica em pacientes com sintomas de dor orofacial e cefaleia tensional. Para tanto, serão selecionados 240 participantes que serão divididos em 4 grupos estratificados por faixa etária. O G1 receberá como tratamento a fotobiomodulação local nos músculos masseter e temporal em 4 sessões, o G2, o tratamento com pulseira que emitirá luz laser na região da artéria radial por 10 minutos em 4 sessões, para o G3 serão realizados os mesmos procedimentos que o G1, mas com o equipamento desligado (sham), e o G4 terá o mesmo procedimento que o G2, porém com o aparelho sem emissão de luz. Antes da aplicação serão coletadas informações sociodemográficas como: idade; fototipo de pele (classificada pela escala de Fitzpatrick), peso, altura, índice de massa corporal (IMC), saturação de oxigênio (SaO2), pressão arterial (PA), frequência cardíaca (FC) e espessura de pele, gordura e músculos da face. Durante a aplicação, coletaremos: temperatura local, SaO2, PA e FC. Antes e após a aplicação do laser, os níveis sanguíneos do lactato e da hemoglobina, a PA e a FC serão mensurados na primeira e na última sessão. Além das variáveis demográficas, antropométricas e hemodinâmicas, será quantificada a energia penetrada por meio de medidor de potência e analisadas as informações dos questionários de sintomas de dor orofacial e cefaleia. Para validação de modelo será utilizada a simulação de monte Carlo a partir das mensurações das variáveis e a transmissão da luz.

Palavras-chave: cefaleia, dor orofacial, dosimetria in vivo, fotobiomodulação, método de Monte Carlo, laser de baixa potência.

Introdução

A dor orofacial foi classificada de acordo com a sua localização em dentária, periodontal, óssea, muscular, nervosa, na mucosa oral, nas glândulas salivares, e na articulação temporomandibular (ATM). Podendo ainda ter manifestações semelhantes a cefaleias primárias, ser de origem idiopática ou estar associada a fatores psicológicos (ansiedade, catastrofização e depressão) e sociais (acesso à assistência médica, estigmas e apoio de familiares e amigos). A dor de origem dentoalveolar e estruturas associadas é o relato mais comum de queixa na região orofacial (Classificação Internacional de Dor Orofacial, 1ª Edição), sendo a dor não odontogênica mais diagnosticada nesta região a de origem da disfunção temporomandibular (DTM) (Häggman-Henrikson B, 2021).

O plano de tratamento para as dores orofaciais deve ser elaborado de acordo com as necessidades do indivíduo. Diferentes terapias podem auxiliar na redução da sensação dolorosa neste quadro. Recomenda-se cautela na escolha de terapias invasivas e tratamentos irreversíveis como terapias de primeira escolha para o tratamento (Rai S et al., 2016).

Dentre os tratamentos não invasivos que têm sido usados para aliviar as condições dolorosas orofaciais estão placas oclusais, ultrassonografia, fisioterapia manual, terapia medicamentosa, exercício oral estimulação elétrica transcutânea e a fotobiomodulação (FBM). A fotobiomodulação com laser de baixa intensidade, tem sido utilizada como tratamento alternativo por se tratar de uma terapia não invasiva e segura relacionada às características de energia e comprimento de onda de baixa intensidade e apresentar respostas anti-inflamatórias, analgésicas e outras respostas biológicas terapêuticas. O mecanismo da FBM pode estar associado à sua influência na síntese, liberação e metabolismo de várias substâncias relacionadas à dor e à analgesia (Aisaiti A et al., 2021).

Na revisão sistemática e meta-análise de Wu X et al., 2021, dos 85 artigos publicados entre os anos de 2009 e 2020, foram avaliados 8 ensaios clínicos randomizados que compararam o tratamento da fotobiomodulação com o grupo placebo em 181 participantes portadores de dor orofacial miofascial. Os resultados foram satisfatórios para o controle da sensação dolorosa, no entanto, os autores destacaram a grande variabilidade de equipamentos e parâmetros dosimétricos. A diferença entre os métodos de escolha de energia, potência e comprimento de onda entre os trabalhos incluídos não permite a elaboração de um protocolo dosimétrico ideal para o tratamento.

Em outra revisão sistemática publicada em 2022 por Oliveira-Souza et al., objetivou determinar quais parâmetros dosimétricos da fotobiomodulação fornecem melhores efeitos na redução da dor em pacientes com dor orofacial. Os autores observaram que os parâmetros de comprimento de onda, energia, tempo e irradiância foram bem diferentes entre os ensaios clínicos incluídos. Os relatos foram do uso de laser de diodo ou gálio-alumínio-arsenieto (GaAlAs), comprimento de onda de 400-800 ou 800-1500 nm, e <25 J/cm^2. Para pacientes com dor articular, laser de diodo e comprimento de onda entre 400 e 800 nm. Para pacientes com dores musculares, os seguintes lasers e parâmetros foram utilizados: laser de diodo, comprimento de onda entre 800 e 1500 nm e de 25 J/cm^2. Para pacientes com dores articulares e musculares, laser infravermelho, comprimento de onda de 800-1500 nm, 100 J/cm ^{2}, e um tempo de aplicação entre 15 e 30 s ou >60 segundos.

A pesquisadora, responsável pelo presente projeto, iniciou os estudos na linha de pesquisa relacionada a DTM e dores orofaciais, com publicações a partir de 2012 nesse tema. O grupo de pesquisas

investigou a relação das dores orofaciais e suas relações com ansiedade, alterações posturais e fatores associados (Motta et al., 2012; Motta et al., 2013; Motta et al., 2015).

A partir de 2013 o grupo, passou a pesquisar o controle da dor na DTM e dores musculares faciais com a aplicação do laser e do LED. Os resultados demonstraram ser satisfatórios com alguns parâmetros (Silva et al.; 2015; Godoy et al., 2017; Viegas et al., 2018; Langella et al., 2018).

Seguindo a linha de atuação, realizou-se ensaio clínico para custo-efetividade do laser no manejo da dor facial. A pesquisa demonstrou que a fotobiomodulação apresentou efeito analgésico e se mostrou mais custo-efetiva em relação ao placebo e placa oclusal (Sobral et.al.; 2018; Sobral et al., 2020).

No ano de 2022 a proponente foi contemplada pelo Edital Edital n° 12/2021 – Programa de Desenvolvimento da Pós-Graduação (PDPG) – Impactos da Pandemia, com o projeto *Efeito Da Laserterapia Transcutânea Sistêmica Versus Local Na Cefaleia Tipo Tensional E Nas Dores Orofaciais Em Indivíduos Pós-Covid-19: Ensaio Clínico Pragmático Randomizado.*

Os ensaios clínicos que avaliam a eficácia da fotobiomodulação têm trazido diferentes conclusões em relação à dosimetria e ainda existe uma inquietação em relação à elaboração dos protocolos, considerando os parâmetros dosimétricos e a justificativa para a escolha de tais parâmetros nos trabalhos publicados (Aisaiti A et al., 2021).

A entrega de energia durante a terapia de fotobiomodulação requer a transmissão de fótons através das camadas externas do tecido, como pele e gordura, antes de atingir o alvo desejado. Acreditamos que a espessura dessas camadas em pacientes com diferentes composições corporais pode interferir nos resultados esperados da terapia. No entanto, esses dados não foram ainda explorados nos ensaios clínicos desse tema.

Com base na necessidade de maior precisão da dosimetria de acordo com as características individuais, levanta-se a hipótese de que as variáveis antropométricas e hemodinâmicas podem ser usadas para determinar os parâmetros apropriados para entregar a energia mais próxima do ideal no controle dado dor orofacial.

Diante disso, o objetivo do presente projeto é avaliar um modelo de simulação de Monte Carlo para prever a distribuição de energia e seus efeitos relacionados às variáveis antropométricas e hemodinâmicas no tratamento de fotobiomodulação com aplicação local e sistêmica em diferentes faixas-etárias.

Espera-se, com o desenvolvimento do presente estudo, desenvolver e validar um modelo de Simulação de Monte Carlo para calcular a dosimetria apropriada para terapias de fotobiomodulação no controle da dor orofacial de acordo com as características físicas e fisiopatológicas de cada indivíduo. Dessa maneira, os profissionais de saúde poderão entregar a energia mais próxima da ideal no tratamento, alcançando os resultados desejados, diminuindo tempo de exposição desnecessária, sub ou sobre tratamento.

Método

Local de estudo e seleção da amostra e aspectos éticos

Trata-se de um estudo clínico de validação de modelo dosimétrico. O teve aprovação do Comitê de Ética em Pesquisa da Universidade Nove de Julho com parecer número 6.080.655. Para os participantes menores de idade será elaborado o termo de assentimento e o termo de consentimento para os respectivos responsáveis. Os procedimentos clínicos de recrutamento, avaliação da elegibilidade e os processos metodológicos serão realizados nos Ambulatórios Integrados de Saúde da UNINOVE – Unidade Vergueiro (São Paulo, SP, Brasil). As técnicas de modelagem de Simulação de Monte Carlo e as análises estatísticas serão realizadas em parceria com o Departamento de Física da Universidade Beira Interior (Covilhã, Portugal).

Amostra

Serão convidados a participar do estudo pessoas saudáveis entre 7 e 65 anos de idade. Optou-se por uma amostra de conveniência, com um total de 240 participantes, sendo 40 participantes de cada faixa etária: (1) 7 a 10 anos; (2) 11 a 14 anos; (3) 15 a 19 anos; (4) 20 a 34 anos; (5) 35 a 49 anos; (6) de 50 a 65 anos.

Critérios de inclusão

- Indivíduos de ambos os sexos, entre 07 e 65 anos

Critérios de exclusão

Serão excluídos os participantes com as seguintes características:

- Gestantes
- Portadores de arritmia
- Portadores de Trombocitopenia
- Portadores de Anemia falciforme
- Portadores de marcapasso
- Pessoas com alteração nos fatores de coagulação

Critérios de descontinuação ou interrupção

Participantes que relatarem qualquer desconforto durante a realização dos procedimentos; que apresentarem sensibilidade à aplicação do laser, serão excluídos da análise e os procedimentos serão interrompidos imediatamente ao relato.

Variáveis do estudo

Serão coletadas informações sociodemográficas como: idade, fototipo de pele (classificada pela escala de Fitzpatrick), peso, altura, saturação de oxigênio, pressão arterial, frequência cardíaca, temperatura local, níveis sanguíneos de lactato e de hemoglobina. Além das variáveis demográficas, antropométricas e hemodinâmicas, será quantificada a energia penetrada, por meio de medidor de potência, e analisadas informações dos questionários de sintomas de dores orofaciais e de cefaleia.

Procedimentos Metodológicos

Coleta dos dados

1. **Lactato Sanguíneo**

O lactato sanguíneo será coletado antes do tratamento, logo após a primeira sessão e após a última sessão. A coleta será realizada na falange distal do dedomédio (uma gota de sangue), após higiene local com álcool a 70%. Para a

punção, a pesquisadora usará luvas cirúrgicas e lancetas descartáveis. A amostra sanguínea será analisada pelo LACTATO DETECT TD-4261 (EcoDiagnóstica, Nova lima, MG, Brasil).

2. **Nível de Hemoglobina**

O nível de hemoglobina será analisado antes do tratamento, logo após a primeirasessão e após a última sessão. Para análise de hemoglobina será utilizada a mesma punção realizada para coleta do lactato. A gota de sangue será analisadapelo Analisador Hb ECO CARE (Eco Diagnóstica, Nova lima, MG, Brasil).

3. **(a) Frequência cardíaca; (b) pressão arterial, (c) saturação de O2; (d) transmissão de luz e (e) temperatura local**

As variáveis frequência cardíaca, pressão arterial, saturação de O2, transmissãode luz e temperatura local serão coletadas durante a aplicação da fotobiomodulação que acontecerá em ambiente privado no consultório odontológico do Núcleo Setorial de Saúde do Centro de Ensino. Para a coleta destas variáveis será utilizado o *Handheld – Vital Sign Monitor*.

(d) Transmissão da luz

A transmissão da luz será avaliada através do dedo indicador e da bochecha (masseter) de cada participante. Será usado o dispositivo SPER *ScientificPocket Laser Power Meter (Scottsdale, AZ, USA)*. O sensor do equipamento seráposicionado na região interna da bochecha ao mesmo tempo em que a pesquisadora estiver aplicando o laser na região do masseter (2 pontos).

(e) Espessura da camada de gordura e músculos da face.

Será utilizado o aparelho BodyMetrix 2000, ultrassom linear com profundidade de 60mm, para avaliar a espessura dos músculos da face. O método é seguro, não emite radiação e não é invasivo. Durante a avaliação, o participante permanecerá em uma posição confortável, e um gel de condução será aplicado na pele sobre o masseter. O aparelho será deslizado no sentido da origem para a inserção do músculo, buscando obter informações precisas. As imagens ultrassonográficas geradas serão posteriormente analisadas usando

o software BodyView, para espessuras das camadas de tecido na região da face.

Intervenções

Grupo 1 – Fotobiomodulação local

O tratamento será realizado em 2 semanas, totalizando 4 sessões (2 sessões por semana) com duração média de 4 minutos por sessão.

Os(As) participantes serão posicionados(as) em cadeira clínica para a aplicação da fotobiomodulação. As intervenções serão realizadas por profissional treinado. O G1 receberá aplicação da fotobiomodulação com o aparelho Therapy EC – DMC, contendo seu comprimento de onda infravermelha de 808 nm e potência de 100 Mw, devidamente calibrado, e energia de 6J por ponto (60 segundos) em2 pontos na região do músculo masseter, 1 ponto no músculo temporal e 1 pontono músculo trapézio na região cervical. A aplicação total levará 4 minutos por sessão.

Nomomento da aplicação, estarão presentes apenas o(a) participante a ser tratado(a), a pesquisadora responsável pelo tratamento e o(a) responsável. Todos(as) utilizarão óculos específicos para proteção ocular. A ponteira do equipamento será desinfectada com álcool a 70% e revestida com plástico transparente descartável (PVC), para evitar contaminações cruzadas, enquanto a limpeza facial prévia do local irradiado será realizada com solução de Clorexidina a 0,2%. Durante as aplicações, o(a) participante permanecerá sentado(a), com o plano de Frankfurt paralelo ao solo. Para complementar as análises de penetração de energia será aplicado 6J no dedo indicador direito.

Grupo 2 – Fotobiomodulação Vascular

A FBMV será aplicada com o mesmo aparelho, usando 660 nm e 100 Mw, e direcionando o feixe de luz para região da artéria radial, durante 10 minutos porsessão. O(A) participante será posicionado(a) sentado(a) em uma cadeira confortávelcom apoio lateral para descanso dos braços durante a aplicação. No momentoda aplicação, estarão presentes somente o(a) participante a ser tratado(a), a pesquisadora responsável pelo tratamento e o(a) responsável. Todos(as)utilizarão óculos específicos para proteção ocular. A parte ativa da pulseira serárevestida com plástico transparente descartável (PVC), evitando contaminaçõescruzadas e, por motivo de higiene, a limpeza prévia do local irradiado serárealizada com solução de Clorexidina a 0,2%. Durante as aplicações o(a)participante permanecerá sentado(a), com o plano de Frankfurt paralelo ao solo.O tratamento será realizado em 2 semanas, totalizando 4 sessões (2 sessões por semana) com duração média de 10 minutos por sessão.

Grupo 3 – Fotobiomodulação local placebo (sham)

O G3 passará pelos mesmos procedimentos descritos para o G1, porém o equipamento emitirá somente o sinal sonoro, exatamente igual ao equipamentoutilizado no G1, sem a emissão de luz laser.

Grupo 4 – Fotobiomodulação vascular placebo

O G4 passará pelos mesmos procedimentos descritos para o G2, porém o equipamento emitirá somente o sinal sonoro, exatamente igual ao equipamentoutilizado no G1, sem a emissão de luz laser.

Os parâmetros dosimétricos dos tratamentos do G1 e G2 são apresentados na Tabela 1.

Tabela 1. Parâmetros dosimétricos de aplicação da fotobiomodulação.

**PARÂMETROS**	**LASER INFRAVERMELHO (Local)**	**LASER VERMELHO (transcutâneo sistêmico)**
Comprimento de onda [nm]	808	660
5t	Contínuo	Contínuo
Potência [mW]	100	100
Diâmetro de abertura [cm]	0,354 (diâmetro do feixe com espaçador)	0,354 cm
Área do feixe [cm^2]	0,0984 (com espaçador)	0,0984 cm^2
Tempo de exposição [s]	60 por ponto	600 s
Fluência [J/cm^2]	61	-
Energia [J]	6 por ponto	60 J
Número de pontos irradiados	4	Sistêmico
Técnica de aplicação	Contato	Contato
Número de sessões	4	4
Frequência de tratamento	2 vezes por semana	2 vezes por semana
Energia total irradiada [J]	96 J	240 J

Validação Modelo de Monte Carlo.

Utilizando a medida de potência de referência, a medida de potência transmitida e a espessura da bochecha , o coeficiente de atenuação efetivo da bochecha será calculado utilizando a Lei de Beer. O coeficiente médio de atenuação efetivo, a espessura média da bochecha e a medida média de potência de referência serão calculados. Serão calculadas as distribuições de taxa de fluência e potência absorvida, bem como a luz transmitida através do meio (transmissão) e absorção multicamadas em um plano paralelo, assumindo luz incidente monocromática. As propriedades ópticas, incluindo os coeficientes de absorção e os coeficientes de espalhamento reduzidos usados nas simulações de Monte Carlo, serão obtidas da literatura e fantomas. As propriedades ópticas da pele serão determinadas na mensuração das variáveis deste estudo. As propriedades ópticas da gordura e do músculo serão medidas pela ultrassonografia.

Análise, gestão dos dados e disseminação

Os dados coletados durante a pesquisa serão armazenados e organizados no repositório Harvard Dataverse (https://dataverse.harvard.edu). Os metadados serão publicados no site no repositório por meio do endereço eletrônico fornecido pela plataforma (DOI).

Os dados referentes aos participantes e aos desfechos da pesquisa permanecerão em sigilo. Somente os pesquisadores terão acesso a essas informações. Após a conclusão da pesquisa, os dados serão publicados e divulgados em eventos e periódicos científicos nacionais e internacionais. Os dados brutos permanecerão na plataforma e após a publicação poderão ser disponibilizados a outros pesquisadores mediante contato com a pesquisadora responsável e mediante acordo de confidencialidade e propriedade intelectual.

Bibliografia:

1. Shah N, Hameed S. Muscle Contraction Tension Headache. 2021 feb 7. In: StatPearls [Internet]. Treasure Island (FL): StatPearls Publishing; 2021 Jan–. PMID: 32965945
2. Leeuw R. Dor orofacial: guia de avaliação, diagnóstico e tratamento. 4ª ed. São Paulo: Quintessence; 2010.
3. Memmedova F, Emre U, Yalın OÖ, Doğan OC. Evaluation of temporomandibular joint disorder in headache patients. Neurol Sci. 2021 Feb 18. doi: 10.1007/s10072-021-05119-z.

4. World Headache Alliance (WHA). Tensión Type Headache, 2016.
5. De Pauw R, Dewitte V, de Hertogh W, Cnockaert E, Chys M, ¿Cagnie B. Consensus among musculoskeletal experts for the management of patients with headache by physiotherapists? A delphi study. Musculoskelet Sci Pract. 2021 Jan 26; 52:102325. doi: 10.1016/j.msksp.2021.102325.
6. Heldarskard GF, Kolding LT, Hvedstrup J, Schytz HW. Myofascial trigger points in migraine and tension-type headache. J Headache Pain. 2018 Sep 10;19(1):84. doi: 10.1186/s10194-018-0913-8.
7. DALEWSKI, Bartosz; KAMIŃSKA, Agata; SZYDłOWSKI, Michał; KOZAK, Małgorzata;
8. SOBOLEWSKA, Ewa. Comparison of Early Effectiveness of Three Different Intervention Methods in Patients with Chronic Orofacial Pain: a randomized, controlled clinical trial.: A Randomized, Controlled Clinical Trial. Pain Research And Management, [s.l.], v. 2019, p. 1-9, 11 mar. 2019.
9. Lippi G, Mattiuzzi C, Bovo C, Henry BM. Headache is an important symptom in patients with coronavirus disease 2019 (COVID-19). Diagnosis (Berl). 2020 Nov 18;7(4):409-411. doi: 10.1515/dx-2020-0048. PMID: 32478675.
10. Martínez-Pías E, García-Azorín D, Trigo-López J, Sierra A, Guerrero-Peral AL. Migraña crónica con cefalea diaria. Revisión de la bibliografía [Chronic migraine with daily headache. Literature review]. Rev Neurol. 2021 Feb 16;72(4):133-140. Spanish. doi: 10.33588/rn.7204.2020583. PMID: 33570160.
11. Rocha-Filho PAS, Magalhães JE. Headache associated with COVID-19: Frequency, characteristics and association with anosmia and ageusia. Cephalalgia. 2020 Nov;40(13):1443-1451. doi: 10.1177/0333102420966770.
12. Medeiros RA, Vieira DL, Silva EVFD, Rezende LVML, Santos RWD, Tabata LF. Prevalence of symptoms of temporomandibular disorders, oral behaviors, anxiety, and depression in Dentistry students during the period of social isolation due to COVID-19. J Appl Oral Sci. 2020 Nov 30;28:e20200445.
13. Asquini G, Bianchi AE, Borromeo G, Locatelli M, Falla D. The impact of Covid-19-related distress on general health, oral behaviour, psychosocial features, disability and pain intensity in a cohort of Italian patients with temporomandibular disorders. PLoS One. 2021 Feb 2;16(2):e0245999. doi: 10.1371/journal.pone.0245999.
14. Almeida-Leite CM, Stuginski-Barbosa J, Conti PCR. How psychosocial and economic impacts of COVID-19 pandemic can interfere on bruxism and temporomandibular disorders? J Appl Oral Sci. 2020;28:e20200263. doi: 10.1590/1678-7757-2020-0263.

15. Uygun Ö, Ertaş M, Ekizoğlu E, Bolay H, Özge A, Kocasoy Orhan E, Çağatay AA, Baykan B. Headache characteristics in COVID-19 pandemic-a survey study. J Headache Pain. 2020 Oct 13;21(1):121. doi: 10.1186/s10194-020-01188-1.
16. Meneguzzo DT, Lopes LA, Pallota R, Soares-Ferreira L, Lopes-Martins RA, Ribeiro MS. Prevention and treatment of mice paw edema by near-infrared low-level laser therapy on lymph nodes. Lasers Med Sci 2013; 28: 973–80. Barretto SR, de Melo GC, dos Santos JC, de Oliveira MG, Pereira-Filho RN, Alves AV, Ribeiro MA, Lima-Verde IB, Quintans Júnior LJ, de Albuquerque-Júnior RL, Bonjardim LR. Evaluation of anti-nociceptive and anti-inflammatory activity of low-level laser therapy on temporomandibular joint inflammation in rodents. J Photochem Photobiol B 2013; 129: 135–42.
17. Núñez SC, França CM, Silva DF, Nogueira GE, Prates RA, Ribeiro MS. The influence of red laser irradiation timeline on burn healing in rats. Lasers Med Sci 2013; 28: 633–41.
18. Alghadir A, Omar MT, Al-Askar AB, Al-Muteri NK. Effect of low-level laser therapy in patients with chronic knee osteoarthritis: a single-blinded randomized clinical study. Lasers Med Sci 2014; 29: 749–55.
19. França CM, França CM, Nuñez SC, Prates RA, Noborikawa E, Faria MR, Ribeiro MS. Low-intensity red laser on the prevention and treatment of induced-oral mucositis in hamsters. J Photochem Photobiol B 2009.
20. Sobral APT, Godoy CLHD, Fernandes KPS, et al .Photomodulation in the treatment of chronic pain in patients with temporomandibular disorder: protocol for cost-effectiveness analysis BMJ Open 2018;8:e018326. doi: 10.1136/bmjopen-2017-018326
21. Magri, L. V., Carvalho, V. A., Rodrigues, F. C. C., Bataglion, C., & Leite-Panissi, C. R. A. (2018). Non-specific effects and clusters of women with painful TMD responders and non-responders to LLLT: double-blind randomized clinical trial. Lasers in medical science, 33(2), 385-392.
22. Borges, R.M. et al. Effects of different photobiomodulation dosimetries on temporomandibular dysfunction: a randomized, double-blind, placebo-controlled clinical trial. Lasers Med Sci,2018; 33(9),1859-1866.
23. Manfredini, D., Favero, L., Cocilovo, F., Monici, M., & Guarda-Nardini, L. A comparison trial between three treatment modalities for the management of myofascial pain of jaw muscles: A preliminary study. CRANIO®, 2017; 36(5), 327-331.
24. Magri LV, Carvalho VA, Rodrigues FC, Bataglion C, Leite-Panissi CR. Effectiveness of low-level laser therapy on pain intensity, pressure pain threshold,

and SF-MPQ indexes of women with myofascial pain. Lasers Med Sci. 2017 Feb;32(2):419-428. doi: 10.1007/s10103-016-2138-x. Epub 2017 Jan 4. PMID: 28054261.
