# Supplementary material for: Dosimetry model for photobiomodulation based on anthropometric and hemodynamic variables in patients with orofacial pain post-Covid-19: Study protocol for randomized clinical trial
Source: PLoS One. 2024 Oct 15;19(10):e0309073. doi: 10.1371/journal.pone.0309073 (PMC11478869; doi:10.1371/journal.pone.0309073)
Supplement: S4 File — (PDF) [file pone.0309073.s004.pdf]

MODEL VALIDATION FOR PHOTOBIOMODULATION DOSIMETRY BASED ON ANTHROPOMETRIC AND HEMODYNAMIC VARIABLES IN PATIENTS WITH OROFACIAL PAIN: study protocol

Summary

Orofacial pain and tension headache are symptoms that affect a large part of the population, compromising productivity, social skills and functional development. Treatment to reduce pain sensation should be chosen with caution, as drug treatment can bring side effects and overload the body of patients with pain. Low-level laser has been used with local and systemic (vascular) applications for pain control. However, there is still a doubt in the literature about the ideal dosimetric parameters for photobiomodulation treatment according to the patient's characteristics. The objective of this project is to validate a model for dosimetry based on the relationship between the effects of photobiomodulation with anthropometric and hemodynamic variables, both in the use with local application and with the systemic application in patients with symptoms of orofacial pain and tension headache. To this end, 240 participants will be selected and divided into 4 groups stratified by age group. G1 will receive as treatment the local photobiomodulation in the masseter and temporal muscles in 4 sessions, G2, the treatment with a bracelet that will emit laser light in the region of the radial artery for 10 minutes in 4 sessions, for G3 the same procedures will be performed as G1, but with the equipment turned off (sham), and G4 will have the same procedure as G2, but with the device not emitting light. Prior to the application, sociodemographic information will be collected, such as: age; skin phototype (classified by the Fitzpatrick scale), weight, height, body mass index (BMI), oxygen saturation (SaO_2), blood pressure (BP), heart rate (HR), and thickness of skin, fat, and facial muscles. During the application, we will collect: local temperature, SaO_2, PA and FC. Before and after laser application, blood levels of lactate and hemoglobin, BP and HR will be measured in the first and last session. In addition to the demographic, anthropometric and hemodynamic variables, the energy penetrated by means of a power meter will be quantified and the information from the questionnaires on orofacial pain and headache symptoms will be analyzed. For model validation, the Monte Carlo simulation will be used from the measurements of the variables and the light transmission.

Keywords: headache, orofacial pain, in vivo dosimetry, photobiomodulation, Monte Carlo method, low-level laser.

Introduction

Orofacial pain was classified according to its location as dental, periodontal, bone, muscular, nervous, oral mucosa, salivary glands, and temporomandibular joint (TMJ). It may also have manifestations similar to primary headaches, be of idiopathic origin or be associated with psychological factors (anxiety, catastrophizing and depression) and social factors (access to medical care, stigma and support from family and friends). Pain of dentoalveolar origin and associated structures is the most common report of complaint in the orofacial region (International Classification of Orofacial Pain, 1st Edition), and non-odontogenic pain most diagnosed in this region is the origin of temporomandibular disorder (TMD) (Häggman-Henrikson B, 2021).

The treatment plan for orofacial pain should be designed according to the needs of the individual. Different therapies can help reduce the painful sensation in this condition. Caution is advised in choosing invasive therapies and irreversible treatments as first-choice therapies for treatment (Rai S et al., 2016).

Among the non-invasive treatments that have been used to relieve painful orofacial conditions are occlusal splints, ultrasound, manual physical therapy, drug therapy, oral exercise, transcutaneous electrical stimulation, and photobiomodulation (FBM). Low-level laser photobiomodulation has been used as an alternative treatment because it is a non-invasive and safe therapy related to low-intensity energy and wavelength characteristics and presents anti-inflammatory, analgesic and other therapeutic biological responses. The mechanism of FBM may be associated with its influence on the synthesis, release, and metabolism of various substances related to pain and analgesia (Aisaiti A et al., 2021).

In the systematic review and meta-analysis by Wu X et al., 2021, of the 85 articles published between 2009 and 2020, 8 randomized clinical trials were evaluated that compared the photobiomodulation treatment with the placebo group in 181 participants with myofascial orofacial pain. The results were satisfactory for the control of pain sensation, however, the authors highlighted the great variability of equipment and dosimetric parameters. The difference between the methods of choosing energy, power and wavelength among the included studies does not allow the elaboration of an ideal dosimetric protocol for the treatment.

In another systematic review published in 2022 by Oliveira-Souza et al., we aimed to determine which dosimetric parameters of photobiomodulation provide better effects on pain reduction in patients with orofacial pain. The authors noted that the parameters of wavelength, energy, time, and irradiance were quite different between the included clinical trials. The reports were of the use of diode or gallium-aluminum-arsenide (GaAlAs) lasers, wavelengths of 400-800 or 800-1500 nm, and <25 $J/^{cm2}$. For patients with joint pain, diode laser and wavelength between 400 and 800 nm. For patients with muscle pain, the following lasers and parameters were used: diode laser, wavelength between 800 and 1500 nm and 25 J/cm2. For patients with joint and muscle pain, infrared laser, wavelength of 800-1500 nm, 100 J/cm2, and an application time between 15 and 30 s or >60 seconds.

The researcher, responsible for the present project, began studies in the line of research related to TMD and orofacial pain, with publications from 2012 onwards on this topic. The research group investigated the relationship between orofacial pain and its relationship with anxiety, postural changes and associated factors (Motta et al., 2012; Motta et al., 2013; Motta et al., 2015).

Starting in 2013, the group began to research pain control in TMD and facial muscle pain with the application of laser and LED. The results proved to be satisfactory with some parameters (Silva et al.; 2015; Godoy et al., 2017; Viegas et al., 2018; Langella et al., 2018).

Following the line of action, a clinical trial was carried out for the cost-effectiveness of laser in the management of facial pain. The research demonstrated that photobiomodulation had an analgesic effect and proved to be more cost-effective compared to placebo and occlusal splint (Sobral et.al.; 2018; Sobral et al., 2020).

In 2022, the proponent was contemplated by the Public Notice No. 12/2021 – Graduate Development Program (PDPG) – Impacts of the Pandemic, with the project *Effect of Systemic versus Local Transcutaneous Laser Therapy on tension-type headache and orofacial pain in post-Covid-19 individuals: Randomized Pragmatic Clinical Trial.*

Clinical trials evaluating the efficacy of photobiomodulation have brought different conclusions regarding dosimetry and there is still concern about the development of protocols, considering dosimetric parameters and the justification for the choice of such parameters in published studies (Aisaiti A et al., 2021).

Energy delivery during photobiomodulation therapy requires the transmission of photons through the outer layers of tissue, such as skin and fat, before reaching the desired target. We believe that the thickness of these layers in patients with different body compositions may interfere with the expected results of therapy. However, these data have not yet been explored in clinical trials on this topic.

Based on the need for greater precision of dosimetry according to individual characteristics, it is hypothesized that anthropometric and hemodynamic variables can be used to determine the appropriate parameters to deliver the closest to optimal energy in the control of orofacial pain.

Therefore, the objective of the present project is to evaluate a Monte Carlo simulation model to predict the energy distribution and its effects related to anthropometric and hemodynamic variables in the treatment of photobiomodulation with local and systemic application in different age groups.

It is expected, with the development of the present study, to develop and validate a Monte Carlo Simulation model to calculate the appropriate dosimetry for photobiomodulation therapies in the control of orofacial pain according to the physical and pathophysiological characteristics of each individual. In this way, health professionals will be able to deliver the energy closest to the ideal in the treatment, achieving the desired results, reducing unnecessary exposure time, under or over treatment.

Method

Place of study and selection of the sample and ethical aspects

This is a clinical study of dosimetric model validation. The study was approved by the Research Ethics Committee of Universidade Nove de Julho under opinion number 6,080,655. For underage participants, a consent form and a consent form will be prepared for their respective guardians. The clinical procedures of recruitment, evaluation of eligibility and methodological processes will be carried out in

the Integrated Health Outpatient Clinics of UNINOVE – Vergueiro Unit (São Paulo, SP, Brazil). The Monte Carlo Simulation modeling techniques and statistical analyses will be carried out in partnership with the Department of Physics of the University of Beira Interior (Covilhã, Portugal).

Sample

Healthy people between 7 and 65 years of age will be invited to participate in the study. A convenience sample was chosen, with a total of 240 participants, 40 participants from each age group: (1) 7 to 10 years; (2) 11 to 14 years old; (3) 15 to 19 years old; (4) 20 to 34 years old; (5) 35 to 49 years old; (6) from 50 to 65 years of age.

Inclusion criteria

- Individuals of both sexes, between 07 and 65 years old

Exclusion Criteria

Participants with the following characteristics will be excluded:

-Pregnant
- Arrhythmia sufferers
- Patients with Thrombocytopenia
- Sickle cell anemia
- Pacemaker carriers
- People with changes in clotting factors

Discontinuation or discontinuation criteria

Participants who report any discomfort during the performance of the procedures; who are sensitive to the application of the laser will be excluded from the analysis and the procedures will be interrupted immediately upon the report.

Study variables

Sociodemographic information will be collected, such as: age, skin phototype (classified by the Fitzpatrick scale), weight, height, oxygen saturation, blood pressure, heart rate, local temperature, blood levels of lactate and hemoglobin. In addition to demographic, anthropometric and hemodynamic variables, the penetrated energy will be quantified by means of a power meter, and information from the orofacial pain and headache symptom questionnaires will be analyzed.

Methodological Procedures

Data collection

1. **Blood Lactate**

Blood lactate will be collected before treatment, just after the first session, and after the last session. The collection will be performed in the distal phalanx of the middle finger (a drop of blood), after local hygiene with 70% alcohol. For the

puncture, the researcher will wear surgical gloves and disposable lancets. The blood

sample will be analyzed by LACTATO DETECT TD-4261 (Eco Diagnóstica, Nova Lima, MG, Brazil).

2. **Hemoglobin Level**

The haemoglobin level will be analysed before treatment, just after the first session and after the last session. For hemoglobin analysis, the same puncture performed for lactate collection will be used. The drop of blood will be analyzed by the Hb ECO CARE Analyzer (Eco Diagnóstica, Nova Lima, MG, Brazil).

3. **(a) Heart rate; (b) blood pressure, (c) O2 saturation; (d) light transmission and (e) local temperature**

The variables heart rate, blood pressure, O2 saturation, light transmission and local temperature will be collected during the application of photobiomodulation, which will take place in a private environment in the dental office of the Health Sector Center of the Teaching Center. To collect these variables, the *Handheld – Vital Sign Monitor will be used*.

(d) Light transmission

Light transmission will be assessed through the index finger and cheek (masseter) of each participant. The SPER *Scientific Pocket Laser Power Meter (Scottsdale, AZ, USA) device will be used*. The equipment's sensor will be positioned in the inner region of the cheek at the same time that the researcher is applying the laser in the masseter region (2 points).

(e) Thickness of the fat layer and muscles of the face.

The BodyMetrix 2000 device, linear ultrasound with a depth of 60mm, will be used to evaluate the thickness of the facial muscles. The method is safe, does not emit radiation, and is non-invasive. During the assessment, the participant will remain in a comfortable position, and a conduction gel will be applied to the skin over the masseter. The device will be slid in the direction of origin for the insertion of the muscle, seeking to obtain accurate information. The generated ultrasound images will be further analyzed using the BodyView software, for thicknesses of the tissue layers in the face region.

Interventions

Group 1 – Local photobiomodulation

The treatment will be carried out in 2 weeks, totaling 4 sessions (2 sessions per week)

with an average duration of 4 minutes per session.
Participants will be positioned in a clinical chair for the application of photobiomodulation. The interventions will be performed by a trained professional. G1 will receive photobiomodulation with the Therapy EC – DMC device, containing its infrared wavelength of 808 nm and power of 100 Mw, properly calibrated, and energy of 6J per point (60 seconds) at 2 points in the masseter muscle region, 1 point in the temporal muscle and 1 point in the trapezius muscle in the cervical region. The full application will take 4 minutes per session.
At the time of application, only the participant to be treated, the researcher responsible for the treatment and the person responsible for the treatment will be present. Everyone will wear specific eyewear for eye protection. The tip of the equipment will be disinfected with 70% alcohol and coated with disposable transparent plastic (PVC), to avoid cross-contamination, while the previous facial cleaning of the irradiated site will be carried out with 0.2% Chlorhexidine solution. During the applications, the participant will remain seated, with the Frankfurt plane parallel to the ground. To complement the energy penetration analyses, 6J will be applied to the right index finger.

Group 2 – Vascular Photobiomodulation

The FBMV will be applied with the same device, using 660 nm and 100 Mw, and directing the light beam to the radial artery region for 10 minutes per session.
The participant will be positioned seated in a comfortable chair with lateral support to rest the arms during the application. At the time of application, only the participant to be treated, the researcher responsible for the treatment and the person responsible for the treatment will be present. Everyone will wear specific eyewear for eye protection. The active part of the bracelet will be coated with disposable transparent plastic (PVC), avoiding cross-contamination and, for hygiene reasons, the previous cleaning of the irradiated site will be carried out with 0.2% Chlorhexidine solution. During the applications, the participant will remain seated, with the Frankfurt plane parallel to the ground. The treatment will be carried out in 2 weeks, totaling 4 sessions (2 sessions per week) with an average duration of 10 minutes per session.

Group 3 – Placebo local photobiomodulation (sham)

The G3 will go through the same procedures described for the G1, but the equipment will only emit the sound signal, exactly the same as the equipment used in the G1, without the emission of laser light.

Group 4 – Placebo vascular photobiomodulation

The G4 will go through the same procedures described for the G2, but the equipment will only emit the sound signal, exactly the same as the equipment used in the G1, without the emission of laser light.

The dosimetric parameters of the G1 and G2 treatments are shown in Table 1.

Table 1. Dosimetric parameters for the application of photobiomodulation.

**PARAMETERS**	**LASER INFRARED (Local)**	**RED LASER (systemic transcutaneous)**
Wavelength [nm]	808	660
5T	Continuous	Continuous
Power [mW]	100	100
Opening diameter [cm]	0.354 (diameter of the beam with spacer)	0.354 cm
Beam area [cm2]	0.0984 (with spacer)	0.0984 cm²
Exposure time[s]	60 per point	600 sec.
Creep [J/cm2]	61	-
Energy [J]	6 per point	60 J
Number of points Irradiated	4	Systemic
Application Technique	Contact	Contact
Number of sessions	4	4
Frequency of treatment	2 times a week	2 times a week
Total radiated energy [J]	96 J	240 J

Monte Carlo Model Validation.

Using the reference power measure, the transmitted power measure, and the cheek thickness, the effective cheek attenuation coefficient will be calculated using Beer's Law. The mean effective attenuation coefficient, mean cheek thickness, and mean reference power measure will be calculated. The distributions of creep rate and absorbed power will be calculated, as well as the light transmitted through the medium (transmission) and multilayer absorption in a parallel plane, assuming monochromatic incident light. The optical properties, including the absorption coefficients and the reduced scattering coefficients used in the Monte Carlo simulations, will be obtained from the literature and phantoms. The optical properties of the skin will be determined in the measurement of the variables of this study. The optical properties of fat and muscle will be measured by ultrasonography.

Analysis, data management and dissemination

The data collected during the research will be stored and organized in the Harvard Dataverse (https://dataverse.harvard.edu) repository. The metadata will be published on the website in the repository through the electronic address provided by the platform (DOI).

Data regarding participants and research outcomes will remain confidential. Only researchers will have access to this information. After the conclusion of the research, the data will be published and disseminated in national and international scientific events and journals. The raw data will remain on the platform and, after publication, may be made available to other researchers by contacting the principal investigator and by means of a confidentiality and intellectual property agreement.

Bibliography:

1. Shah N, Hameed S. Muscle Contraction Tension Headache. 2021 Feb 7. In: StatPearls [Internet]. Treasure Island (FL): StatPearls Publishing; 2021 Jan–. PMID: 32965945
2. Leeuw R. Orofacial pain: a guide to evaluation, diagnosis, and treatment. 4th ed. São Paulo: Quintessence; 2010.
3. Memmedova F, Emre U, Yalın OÖ, Doğan OC. Evaluation of temporomandibular joint disorder in headache patients. Neurol Sci. 2021 Feb 18. DOI: 10.1007/S10072-021-05119-Z.
4. World Headache Alliance (WHA). Tensión Type Headache, 2016.

5. De Pauw R, Dewitte V, de Hertogh W, Cnockaert E, Chys M, ¿Cagnie B. Consensus among musculoskeletal experts for the management of patients with headache by physiotherapists? A delphi study. Musculoskelet Sci Pract. 2021 Jan 26; 52:102325. doi: 10.1016/j.msksp.2021.102325.
6. Heldarskard GF, Kolding LT, Hvedstrup J, Schytz HW. Myofascial trigger points in migraine and tension-type headache. J Headache Pain. 2018 Sep 10; 19(1):84. DOI: 10.1186/S10194-018-0913-8.
7. DALEWSKI, Bartosz; KAMIŃSKA, Agatha; SZYDŁOWSKI, Michał; KOZAK, Małgorzata;
8. SOBOLEWSKA, Ewa. Comparison of Early Effectiveness of Three Different Intervention Methods in Patients with Chronic Orofacial Pain: a randomized, controlled clinical trial.: A Randomized, Controlled Clinical Trial. Pain Research And Management, [s.l.], v. 2019, p. 1-9, 11 mar. 2019.
9. Lippi G, Mattiuzzi C, Bovo C, Henry BM. Headache is an important symptom in patients with coronavirus disease 2019 (COVID-19). Diagnosis (Berl). 2020 Nov 18; 7(4):409-411. doi: 10.1515/dx-2020-0048. PMID: 32478675.
10. Martínez-Pías E, García-Azorín D, Trigo-López J, Sierra A, Guerrero-Peral AL. Revisión de la bibliografía [Chronic migraine with daily headache. Literature review]. Rev Neurol. 2021 Feb 16; 72(4):133-140. Spanish. DOI: 10.33588/RN.7204.2020583. PMID: 33570160.
11. Rocha-Filho PAS, Magalhães JE. Headache associated with COVID-19: Frequency, characteristics and association with anosmia and ageusia. Cephalalgia. 2020 Nov; 40(13):1443-1451. doi: 10.1177/0333102420966770.
12. Medeiros RA, Vieira DL, Silva EVFD, Rezende LVML, Santos RWD, Tabata LF. Prevalence of symptoms of temporomandibular disorders, oral behaviors, anxiety, and depression in Dentistry students during the period of social isolation due to COVID-19. J Appl Oral Sci. 2020 Nov 30; 28:e20200445.
13. Asquini G, Bianchi AE, Borromeo G, Locatelli M, Falla D. The impact of Covid-19-related distress on general health, oral behaviour, psychosocial features, disability and pain intensity in a cohort of Italian patients with temporomandibular disorders. PLoS One. 2021 Feb 2; 16(2):e0245999. doi: 10.1371/journal.pone.0245999.
14. Almeida-Leite CM, Stuginski-Barbosa J, Conti PCR. How psychosocial and economic impacts of COVID-19 pandemic can interfere on bruxism and temporomandibular disorders? J Appl Oral Sci. 2020; 28:e20200263. doi: 10.1590/1678-7757-2020-0263.

15. Uygun Ö, Ertaş M, Ekizoğlu E, Bolay H, Özge A, Kocasoy Orhan E, Çağatay AA, Baykan B. Headache characteristics in COVID-19 pandemic-a survey study. J Headache Pain. 2020 Oct 13; 21(1):121. DOI: 10.1186/S10194-020-01188-1.
16. Meneguzzo DT, Lopes LA, Pallota R, Soares-Ferreira L, Lopes-Martins RA, Ribeiro MS. Prevention and treatment of mice paw edema by near-infrared low-level laser therapy on lymph nodes. Lasers Med Sci 2013; 28: 973–80. Barretto SR, de Melo GC, dos Santos JC, de Oliveira MG, Pereira-Filho RN, Alves AV, Ribeiro MA, Lima-Verde IB, Quintans Júnior LJ, de Albuquerque-Júnior RL, Bonjardim LR. Evaluation of anti-nociceptive and anti-inflammatory activity of low-level laser therapy on temporomandibular joint inflammation in rodents. J Photochem Photobiol B 2013; 129: 135–42.
17. Núñez SC, França CM, Silva DF, Nogueira GE, Prates RA, Ribeiro MS. The influence of red laser irradiation timeline on burn healing in rats. Lasers Med Sci 2013; 28: 633–41.
18. Alghadir A, Omar MT, Al-Askar AB, Al-Muteri NK. Effect of low-level laser therapy in patients with chronic knee osteoarthritis: a single-blinded randomized clinical study. Lasers Med Sci 2014; 29: 749–55.
19. France CM, France CM, Nuñez SC, Prates RA, Noborikawa E, Faria MR, Ribeiro MS. Low-intensity red laser on the prevention and treatment of induced-oral mucositis in hamsters. Photochem J Photobiol B 2009.
20. Sobral APT, Godoy CLHD, Fernandes KPS, et al . Photomodulation in the treatment of chronic pain in patients with temporomandibular disorder: protocol for cost-effectiveness analysis BMJ Open 2018; 8:e018326. DOI: 10.1136/BMJOPEN-2017-018326
21. Magri, L. V., Carvalho, V. A., Rodrigues, F. C. C., Bataglion, C., & Leite-Panissi, C. R. A. (2018). Non-specific effects and clusters of women with painful TMD responders and non-responders to LLLT: double-blind randomized clinical trial. Lasers in medical science, 33(2), 385-392.
22. Borges, R.M. et al. Effects of different photobiomodulation dosimetries on temporomandibular dysfunction: a randomized, double-blind, placebo-controlled clinical trial. Lasers Med Sci,2018; 33(9),1859-1866.
23. Manfredini, D., Favero, L., Cocilovo, F., Monici, M., & Guarda-Nardini, L. A comparison trial between three treatment modalities for the management of myofascial pain of jaw muscles: A preliminary study. CRANIO,® 2017; 36(5), 327-331.
24. Magri LV, Carvalho VA, Rodrigues FC, Bataglion C, Leite-Panissi CR. Effectiveness of low-level laser therapy on pain intensity, pressure pain threshold,

and SF-MPQ indexes of women with myofascial pain. Lasers Med Sci. 2017 Feb; 32(2):419-428. DOI: 10.1007/S10103-016-2138-X. Epub 2017 Jan 4. PMID: 28054261.
